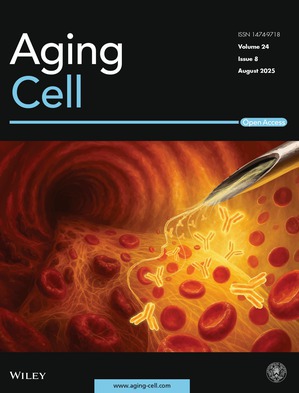# Additional Cover

**DOI:** 10.1111/acel.70201

**Published:** 2025-08-12

**Authors:** Matias Fuentealba, Dobri Kiprov, Kevin Schneider, Wei‐Chieh Mu, Prasanna Ashok Kumaar, Herbert Kasler, Jordan B. Burton, Mark Watson, Heather Halaweh, Christina D. King, Zehra Stara Yüksel, Chelo Roska‐Pamaong, Birgit Schilling, Eric Verdin, David Furman

## Abstract

Cover legend: The cover image is based on the article *Multi‐Omics Analysis Reveals Biomarkers That Contribute to Biological Age Rejuvenation in Response to Single‐Blinded Randomized Placebo‐Controlled Therapeutic Plasma Exchange* by Matias Fuentealba et al., https://doi.org/10.1111/acel.70103.